# Tyrosine 601 of *Bacillus subtilis* DnaK Undergoes Phosphorylation and Is Crucial for Chaperone Activity and Heat Shock Survival^‡^

**DOI:** 10.3389/fmicb.2016.00533

**Published:** 2016-04-19

**Authors:** Lei Shi, Vaishnavi Ravikumar, Abderahmane Derouiche, Boris Macek, Ivan Mijakovic

**Affiliations:** ^1^Division of Systems and Synthetic Biology, Department of Biology and Biological Engineering, Chalmers University of TechnologyGothenburg, Sweden; ^2^Proteome Center Tübingen, Interfaculty Institute for Cell Biology, University of TübingenTübingen, Germany

**Keywords:** quantitative phosphoproteomics, bacterial protein-tyrosine kinases, molecular chaperones, protein folding, protein phosphorylation, SILAC

## Abstract

In order to screen for cellular substrates of the *Bacillus subtilis* BY-kinase PtkA, and its cognate phosphotyrosine-protein phosphatase PtpZ, we performed a triple Stable Isotope Labeling by Amino acids in Cell culture-based quantitative phosphoproteome analysis. Detected tyrosine phosphorylation sites for which the phosphorylation level decreased in the Δ*ptkA* strain and increased in the Δ*ptpZ* strain, compared to the wild type (WT), were considered as potential substrates of PtkA/PtpZ. One of those sites was the residue tyrosine 601 of the molecular chaperone DnaK. We confirmed that DnaK is a substrate of PtkA and PtpZ by *in vitro* phosphorylation and dephosphorylation assays. *In vitro*, DnaK Y601F mutant exhibited impaired interaction with its co-chaperones DnaJ and GrpE, along with diminished capacity to hydrolyze ATP and assist the re-folding of denatured proteins. *In vivo*, loss of DnaK phosphorylation in the mutant strain *dnaK* Y601F, or in the strain overexpressing the phosphatase PtpZ, led to diminished survival upon heat shock, consistent with the *in vitro* results. The decreased survival of the mutant *dnaK* Y601F at an elevated temperature could be rescued by complementing with the WT *dnaK* allele expressed ectopically. We concluded that the residue tyrosine 601 of DnaK can be phosphorylated and dephosphorylated by PtkA and PtpZ, respectively. Furthermore, Y601 is important for DnaK chaperone activity and heat shock survival of *B. subtilis*.

## Introduction

DnaK belongs to the Hsp70 family of molecular chaperones, and plays a ubiquitous and crucial cellular role. For example, it assists protein folding, translocation, assembly and disassembly, and participates in protein quality control (Bukau and Horwich, 1998; Mayer and Bukau, 2005). DnaK comprises of two main domains: an N-terminal nucleotide-binding domain that hydrolizes ATP, and a C-terminal substrate-binding domain, which binds and assists the folding of substrate proteins (Bertelsen et al., 2009; Ahmad et al., 2011). Recently, a DXXXEEV motif in C-terminus of DnaK was identified and suggested to play an important role to maintain the *in vitro* chaperone activity and *in vivo* cell survival upon heat shock in *Escherichia coli* (Gao et al., 2012). For its full activity, DnaK requires the participation of its co-chaperone proteins, DnaJ and GrpE (Schröder et al., 1993). DnaJ stimulates the ATPase activity of DnaK and attracts the unfolded proteins to the active site of DnaK, while GrpE facilitates the ADP–ATP exchange and triggers the folded protein release and resetting of DnaK (Laufen et al., 1999; Mally and Witt, 2001). It was suggested by a previous study that the C-terminus of DnaK is involved in the interaction with DnaJ (Smock et al., 2011). Phosphorylation has been suggested to regulate the activity of DnaK. *E. coli* DnaK gets phosphorylated during heat shock, and the phosphorylated fraction of DnaK exhibits a dramatically increased affinity for unfolded proteins (Sherman and Goldberg, 1993). Moreover, *E. coli* DnaK was found to be serine-phosphorylated during normal growth, and threonine-phosphorylated during MI3 phage infection *in vivo* (Rieul et al., 1987). However, the exact phosphorylated residues were not reported in those studies. It had been previously reported that *E. coli* DnaK can perform autophosphorylation at the residue T199 *in vitro* (Zylicz et al., 1983; McCarty and Walker, 1991), and its capability to hydrolyze ATP was nearly abolished in non-phosphorylatable mutant DnaK T199A (Barthel et al., 2001). Besides T199, recent phosphoproteomics studies in *E. coli* revealed multiple phosphorylation sites in DnaK: S274, S453, S504, T611, and S617 (Macek et al., 2008; Soares et al., 2013). However, the functional relevance of these phosphorylation events still remains unknown.

In *B. subtilis*, DnaK was observed to be phosphorylated during both exponential growth and stress/starvation conditions (Eymann et al., 2007). However, no specific DnaK phosphorylation site has been identified. Here, we report that *B. subtilis* DnaK gets phosphorylated at the residue Y601 by a bacterial protein-tyrosine kinase PtkA (Mijakovic et al., 2003), and dephosphorylated by the phosphotyrosine-protein phosphatase PtpZ (Mijakovic et al., 2005). The relationship between DnaK and the kinase/phosphatase pair was revealed by a global triple SILAC (Stable Isotope Labeling by Amino acids in Cell culture) -based quantitative phosphoproteomics screening, a method we have previously successfully used to detect substrates of bacterial serine/threonine kinases (Ravikumar et al., 2014). *B. subtilis* PtkA is known to function as a signal integration device, and connect a number of cellular processes via protein-substrate phosphorylation (Mijakovic and Deutscher, 2015). Protein substrates that have their activity or cellular localization controlled by PtkA-dependent phosphorylation include single-stranded DNA-binding proteins (Mijakovic et al., 2006), transcription regulators (Derouiche et al., 2013, 2015) and a number of metabolic enzymes (Mijakovic et al., 2003; Jers et al., 2010). PtkA belongs to the family of bacterial tyrosine kinases (BY-kinases), which are known to have relaxed substrate specificity, and a propensity to evolve new kinase-substrate pairs during the process of adaptive evolution (Shi et al., 2014a). Here, we show that tyrosine 601 of DnaK is important for the maintenance of the DnaK chaperone function. The DnaK mutant with tyrosine 601 was replaced by phenylalanine exhibited lower ATP hydrolysis and protein refolding activity, and impaired interaction with its co-chaperones DnaJ and GrpE *in vitro. In vivo*, the mutation compromised the survival of *B. subtilis* upon heat shock.

## Materials and Methods

### SILAC Labeling of Bacterial Cells

Stable Isotope Labeling by Amino acids in Cell culture minimal medium consisting of 15 mM (NH_4_)_2_SO_4_, 2 mM CaCl_2_, 1 μM FeSO_4_.7H_2_O, 8 mM MgSO_4_, 10 μM MnSO_4_, 27 mM KCl, 0.6 mM KH_2_PO_4_, 7 mM C_6_H_5_Na_3_O_7_⋅2H_2_O (Merck), 50 mM Tris-HCl pH 7.5 (Sigma–Aldrich) supplemented with 0.5% glucose (AppliChem), 0.67 mM glutamic acid (Merck) and 490 μM tryptophan (Sigma–Aldrich), was used to grow a lysine auxotrophic strain of *B. subtilis* 168 (also referred to as the wild-type or WT). For labeling purposes, the minimal medium was supplemented with 0.025% of the respective isotopically labeled L-lysine – Light, Lys0: ^12^C_6_^14^N_2_ (Sigma–Aldrich); Medium, Lys4: 4,4,5,6-D_4_; Heavy, Lys8: ^13^C_6_^15^N_2_ (Euriso-Top). An overnight culture grown until an OD_600_ of 0.5–0.6 was used as a pre-inoculum for the main cultures which were grown at 37°C at 200 rpm and harvested at either the late stationary phase of growth or mid-logarithmic phase of growth. The Δ*lysA* WT strain was labeled with “Light” lysine, the Δ*ptkA*Δ*lysA* (kinase) strain with “Medium” lysine and the Δ*ptpZ*Δ*lysA* (phosphatase) strain with “Heavy” lysine. Cells were harvested by centrifugation at 7000 × *g* for 10 min. **Supplementary Figure 1** depicts the entire (phospho) proteomics workflow employed. A total of four biological replicates were performed for the phosphoproteomics experiments and three biological replicates and the whole proteome analysis.

**FIGURE 1:**
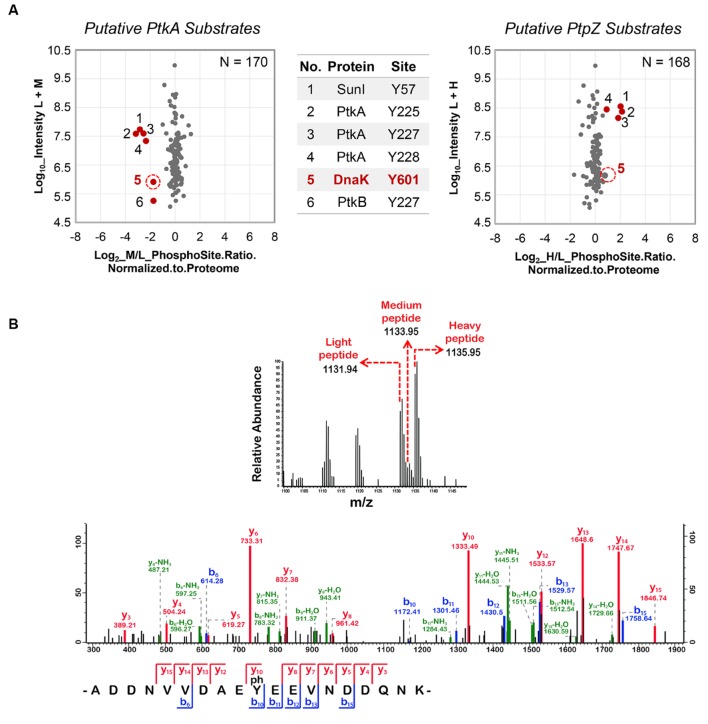
**Stable Isotope Labeling by Amino acids in Cell culture (SILAC) screen: DnaK phosphorylation at Y601 depends on PtkA and PtpZ.** **(A)** Scatter plots showing Δ*ptkA*/WT and Δ*ptpZ*/WT SILAC ratios, respectively. Log_2_ ratios of the phosphorylation sites are normalized to the corresponding protein and plotted against peptide intensities in the log_10_ scale. Significantly changing (*p* = 0.05) SILAC ratios marked in red present potential substrates of PtkA (M/L Ratios) and PtpZ (H/L Ratios), respectively. The spot corresponding to DnaK Y601 is marked with a red circle. **(B)** Spectrum of the DnaK peptide containing phosphorylation at Y601. The SILAC triplet of the DnaK peptide obtained during the survey scan is shown above. The MS/MS spectrum of the DnaK peptide depicting the phosphorylation event on Y601 is shown below.

### Protein Extraction and Digestion

The cell pellet was re-suspended in 1 mL Y-PER Reagent (Thermo Scientific) along with 50 μg mL^-1^ of lysozyme (Sigma–Aldrich), 5 mM of phosphatase inhibitors (sodium fluoride and glycerol-2-phosphate (Sigma–Aldrich)) and protease inhibitor cocktail (Roche). Cell lysis was carried out by incubation at 37°C for 20 min followed by sonication for 30 s at 40% amplitude in order to degrade DNA. Centrifugation was carried out subsequently in order to remove cell debris. Chloroform/methanol precipitation was employed to clean up the crude protein extract followed by dissolution in denaturation buffer containing 6 M urea and 2 M thiourea in 10 mM Tris-HCl pH 8.0. Protein concentration was measured by Bradford assay (Bio-Rad Laboratories). As a quality control step Lys4 and Lys8 labeled protein samples were analyzed for levels of SILAC label incorporation by digesting 10 μg of the labeled protein extract (as described below) and analyzed on the mass spectrometer (MS). Only an incorporation of 98% and above was accepted and taken further for additional analysis.

The following protocol was employed for each replicate triple-SILAC experiment conducted. Protein extracts labeled with Lys0, Lys4, and Lys8 were mixed in equal (1:1:1) ratios to a total of 12 mg, followed by in-solution digestion. In-solution digestion was carried out as described previously (Macek et al., 2007) with slight modifications. The protein extract was reduced with 1 mM dithiothreitol (DTT) for 1 h followed by alkylation with 5.5 mM iodoacetamide (IAA) in the dark for 1 h, at room temperature (RT). Predigestion was carried out with endoproteinase Lys-C (Wako; 1:100, w/w) for 3 h followed by overnight digestion at RT with Lys-C (1:100, w/w) after a dilution of 4 times the volume with deionized water. 100 μg of the resulting peptide mixture was taken for proteome analysis by isoelectric focusing, while the rest was acidified to a pH of 2.5 with 10% trifluoroacetic acid (TFA) and subjected to phosphopeptide enrichment strategies.

### Protein Fractionation by Offgel Isoelectric Focusing

In-solution digested peptides were separated based on differences in their isoelectric point using the 3100 Offgel Fractionator (Agilent Technologies). Peptides were separated into 12 fractions using 13 cm Immobiline Drystrips (pH gradient 3–10; GE Healthcare) using default settings of a maximum current of 50 μA and potential difference of 20 kVh. Fractionated peptides were acidified using 30% acetonitrile (ACN), 5% acetic acid, and 10% TFA. Fractionated peptides were further purified by using C_18_ stage-tips (Ishihama et al., 2006). Briefly, C_18_ (Empore^TM^) stage tips were activated with 100 μL methanol and equilibrated with 200 μL solvent A^∗^ (2% ACN/1% TFA). This was followed by sample loading and washing with 200 μL solvent A (0.5% acetic acid). Peptides were eluted in 50 μL solvent B (80% ACN/0.5% acetic acid), concentrated in a vacuum centrifuge and subjected to nano-LC-MS/MS measurements on the LTQ-Orbitrap Elite (Thermo Fisher Scientific).

### In Gel Digestion (GeLC)

Protein fractionation was also carried out using polyacrylamide gel electrophoresis. Proteins were loaded and separated on a NuPage Bis-Tris 4–12% gradient gel (Invitrogen) based on the manufacturer’s instructions. The gel was stained with Coomassie Blue and cut into 16 slices. Resulting gel slices were destained by three washes with 10 mM ammonium bicarbonate (ABC) and ACN (1:1, v/v) followed by reduction with 10 mM DTT in 20 mM ABC for 45 min at 56°C. Subsequent alkylation was carried out with 55 mM IAA in 20 mM ABC for 30 min at RT in the dark followed by two washes with 5 mM ABC and once with 100% ACN. The gel slices were next dehydrated in a vacuum centrifuge. Proteins were digested with Lys-C (Wako; 12.5 ng μL^-1^ in 20 mM ABC) at 25°C overnight. Resulting peptides were extracted in a three step procedure: Step 1- with 3% TFA in 30% ACN; step 2- with 0.5% acetic acid in 80% ACN; step 3- with 100% ACN. Samples were evaporated in a vacuum centrifuge and peptides were desalted using stage-tips (as described before) and subjected to nano-LC-MS/MS measurements on the LTQ-Orbitrap Elite (Thermo Fisher Scientific).

### Phosphopeptide Enrichment

The 11.8 mg of in-solution digested proteins were subjected to phosphopeptide enrichment in two consecutive steps. In the first step, digested peptides were separated by SCX (Strong Cationic Exchange; Macek et al., 2007). The sample was loaded onto a 1 mL Resource S column (GE Healthcare) in 5 mM KH_2_PO_4_, 30% ACN, and 0.1% TFA (buffer pH 2.7; Merck) with a flow rate of 1 mL min^-1^. A linear salt gradient of 0-35% of 350 mM KCl, 5 mM KH_2_PO_4_, 30% ACN, and 0.1% TFA (buffer pH 2.7) over 30 min was applied to elute the bound peptides, resulting in sixteen 2 mL fractions. Multiply phosphorylated peptides that do not bind to the column remain in the flow-through and hence that was collected separately. The 16 SCX fractions were pooled together according to estimated peptide amounts to form a total of nine fractions on which a second stage of phosphopeptide enrichment was performed, along with the flow-through, using TiO_2_ (Titanium dioxide) chromatography (Macek et al., 2008). TiO_2_ beads of 10 μm (MZ Analysentechnik) were resuspended in a solution containing 2,5-dihydrobenzoic acid in 80% ACN (final concentration 30 mg mL^-1^). 5 mg of the TiO_2_ bead slurry was added to each of the fractions including the flow-through fraction and incubated for 30 min at RT by end-over-end rotation. The TiO_2_ enrichment was repeated twice for each of the pooled SCX fractions and five times in case of the flow-through. After incubation, the beads were washed once with 1 mL of 30% ACN/80% TFA and a second time with 80% ACN/0.1% TFA, for 10 min each. The bound phosphopeptides were eluted from the beads with 100 μL of 40% NH_4_OH solution in 60% ACN, pH > 10.5. The elution step was repeated for a total of three times. The sample volume was reduced in a vacuum centrifuge at RT, acidified to a pH of 1.5 and desalted using C_18_ stage-tips (as before) and subjected to nano-LC-MS/MS measurements on the LTQ-Orbitrap XL (Thermo Fisher Scientific).

Additionally, 20 mg of digested peptides were separately enriched specifically for phosphorylated tyrosines using the Phospho-Tyrosine antibody (PTMScan^®^, Cell Signaling) according to the manufacturer’s instructions with slight modifications. Briefly, the digested peptides were acidified (as before) and desalted by solid phase extraction. A C_18_ cartridge (Sep-Pak Classic, Waters) was activated with 5 mL methanol followed by equilibration with 5 mL solvent A^∗^ (2% ACN/1% TFA). The sample to be desalted was next run through the column. This was followed by a wash step with 5 mL solvent A (0.5% acetic acid) and finally elution with 6 mL of 80% ACN/6% TFA. The eluate was evaporated in a vacuum centrifuge and taken for enrichment with the pTyr antibody. The antibody bead slurry was washed four times with 1x PBS (phosphate buffered saline). The eluate from the solid phase extraction procedure was mixed with the antibody slurry and incubated at 4°C for 2 h by end-over-end rotation. The pTyr peptides bound to the antibody beads were transferred to spin columns (Sigma–Aldrich) and washed twice with IAP (immunoaffinity purification) buffer (50 mM MOPS, 10 mM sodium phosphate pH 7.2, 50 mM sodium chloride). This was followed by three washes with Milli-Q water and elution twice with 0.1% TFA. The eluate was stage-tipped as before and injected on the LTQ-Orbitrap Elite (Thermo Fisher Scientific).

### Mass Spectrometric Analysis

Samples were measured on an Easy-LC nano-HPLC (Proxeon Biosystems) coupled to an LTQ-Orbitrap XL or an LTQ-Orbitrap Elite MS, as described previously (Franz-Wachtel et al., 2012; Krug et al., 2013). Chromatographic separation was done on an in-house packed 15 cm fused silica emitter with reversed-phase ReproSil-Pur C18-AQ 3 μm resin (Dr. Maisch GmbH), with an inner diameter of 75 μm and a tip diameter of 8 μm. Peptides were injected onto the column with solvent A at a flow rate of 500 nL min^-1^ and a pressure of 280 bars. Peptides were eluted at a constant flow rate of 200 nL min^-1^ using a segmented gradient (130 min on the Orbitrap XL; 90 min or 230 min on the Orbitrap Elite) of 5–90% solvent B. Separated peptides were ionized by ESI (Proxeon Biosystems). The mass spectrometer was operated on a data-dependent mode. Survey full-scans for the MS spectra were recorded between 300 and 2000 Thompson at a resolution of 60,000 (on the Orbitrap XL) or 120,000 (on the Orbitrap Elite) with a target value of 10^6^ charges in the Orbitrap mass analyzer. The top five (on the Orbitrap XL) or top 20 (on the Orbitrap Elite) most intense peaks from the survey scans were selected for fragmentation with collision induced dissociation (CID) with a target value of 5000 charges in the linear ion trap analyzer in each scan cycle. For phosphoproteomics analysis, ions were fragmented by multi stage activation with neutral loss occurring at –97.97, –48.98, and –32.66. Dynamic exclusion was set at 90 s. In addition, the lock masses option was enabled on the Orbitrap-XL for internal calibration (Olsen et al., 2005).

### Data Processing and Bioinformatic Analysis

Acquired MS spectra were processed with MaxQuant software package version 1.5.1.0 (Cox et al., 2009, 2011), integrated with the Andromeda search engine. Database search was performed against a target-decoy database of *B. subtilis* 168 obtained from Uniprot (taxonomy ID 1423), containing 4,195 *B. subtilis* protein entries and 248 commonly observed laboratory contaminant proteins. Endoprotease Lys-C was specified as the protease with a maximum missed cleavage of two. Three isotopic forms of lysine (light, medium, and heavy) were specified in the search space. Oxidation of methionines, N-terminal acetylation, and phosphorylation on serine, threonine, and tyrosine residues was specified as a variable modification. Initial maximum allowance for mass tolerance was set to six ppm for the survey scan and 0.5 Da for CID fragment ions. Carbamidomethylation on cysteines was set as a fixed modification. Re-quantify was enabled. A false discovery rate of 1% was applied at the peptide, protein and phosphorylated site level individually. Fragments with a minimum length of seven amino acids were used for SILAC peptide quantification. Phosphorylation events with a localization probability of ≥0.75 were considered localized on the respective S/T/Y residue. A posterior error probability (PEP) score filter of ≤0.001 was applied for the modified peptides. MS/MS spectra of phosphorylated peptides were manually validated for good *b*- and *y*- ion series coverage using MaxQuant Viewer (version 1.5.1.0). Ratios from each SILAC experiment are relative to the Light labeled WT (control) strain. The SILAC ratios of the phosphorylation sites were further normalized to the respective protein ratios in order to eliminate a bias due to changing protein abundance. Phosphorylation sites were considered as differentially regulated based on “Significance B” (*p* = 0.05; Cox et al., 2009) calculated after normalization with the corresponding protein ratios.

### Strain Construction and Growth Condition

Cells were routinely grown in LB medium, with addition of tetracycline (8 μg ml^-1^), ampicillin (100 μg ml^-1^) for *E. coli*, tetracycline (15 μg ml^-1^), spectinomycin (60 μg ml^-1^), phleomycin (2 μg ml^-1^), neomycin (5 μg ml^-1^) for *B. subtilis*, when needed. *E. coli* strain nm522 was used for plasmid construction, NEB Express *I*^q^ Competent *E. coli* was used for heterologous protein purification. Oligos listed in **Supplementary Table 2** were used to amplify respective DNA fragments from *B. subtilis* 168 genomic DNA by PCR. For SILAC- quantitative phosphoproteome analysis, *B. subtilis* Δ*ptkA* and Δ*ptpZ* were constructed from *B. subtilis* Δ*lysA* (Ravikumar et al., 2014) through the pG^+^host system (Maguin et al., 1996; Monedero et al., 2001). The pG^+^host8 vector is tetracycline resistance and carries a thermosensitive replicon that replicates at 28°C but not at 37°C. The upstream and downstream fragments of *ptkA* ORF were obtained by oligo pairs ptkAko_1 and ptkAko_2, ptkAko_3 and ptkAko_4 (**Supplementary Table 2**), and subsequently inserted into pG^+^host8 vector. The resulting plasmid was transformed into *B. subtilis* Δ*lysA* and selected by tetracycline at 37°C. The obtained strain was grown in LB without tetracycline in order to allow the pop-out of pG^+^host8 vector. The same strategy was employed to construct *B. subtilis* Δ*ptpZ* by using the oligo pairs ptpZko_1 and ptpZko_2, ptpZko_3 and ptpZko_4. Construction of the *B. subtilis dnak* Y601F mutant at the natural *dnak* locus was performed using the modified mutation delivery method described previously (Fabret et al., 2002). Briefly, the partially overlapping DNA fragments containing parts of the *dnaK* Y601F gene and the flanking regions were obtained by the oligo pairs dnaKF_1/dnaKF_4 and dnaKF_3/dnaKF_2. Using primers dnaKF_1 and dnaKF_2, these fragments were PCR-joined to the insertion cassette containing the phage lambda cI repressor gene and the phleomycin resistance marker. The resulting PCR products were used to transform the competent *B. subtilis* strain TF8A Pr-neo::Δ*aupp*, in which the neomycin resistance gene is expressed from the lambda promoter negatively controlled by the cI repressor. The transformants were selected for phleomycin resistance and neomycin sensitivity. Finally, counter-selection for neomycin resistance and phleomycin sensitivity was applied to select clones which had lost the insertion cassette from the chromosome via recombination between the flanking direct repeats. To construct *B. subtilis ptpZ* overexpression strain, *gfp* in pSG1729 was replaced by *ptpZ* ORF through KpnI and XhoI. *ptpZ* ORF was obtained by the oligo pairs ptpZ_1729_fwd and ptpZ_1729_rev. The resulting plasmid was used to transform BS33. A similar strategy was employed to construct *dnaK* complementation strain for *B. subtilis dnaK* Y601F. *dnaK* ORF was amplified by the oligos dnak_1729_fwd and dnak_1729_rev. The obtained plasmid was transformed into *B. subtilis dnaK* Y601F. To remove *gfp* from pSG1729, pSG1729 was digested by KpnI and XhoI, the two sticky ends were then made blunt and ligated. The resulting plasmid was transformed into *dnaK* Y601F and WT to obtain the control strain.

### Synthesis and Purification of Affinity-Tagged Protein

*dnaK* WT, *dnaK* Y601F, *dnaJ*, and *grpE* were amplified by using the oligo pairs DnaK_fwd and DnaK_rev, DnaK_fwd and DanK_601F_rev, DnaJ_fwd and DnaJ_rev, grpE_fwd and grpE_rev (**Supplementary Table 2**) from *B. subtilis* 168 genomic DNA. All PCR fragments were cloned in pQE30 (Qiagen) to obtain 6xHis-tag fusion proteins. Strep-tagged versions of proteins were obtained using a pQE-30 vector in which the His-tag was replaced by strep-tag (Jers et al., 2010). Protein synthesis and purification were performed as described previously (Mijakovic et al., 2003). In brief, protein induction was carried out when the culture reached an OD_600_ of 0.6 by adding 1 mM IPTG. Cells were harvested 3 h later and disrupted by sonication. The 6xHis- or Strep-tagged proteins were purified from crude extracts by Ni-NTA (Qiagen), or Strep Tactin affinity chromatography (Novagen), respectively. Purified proteins were desalted on PD-10 columns (GE Healthcare).

### Circular Dichroism Spectroscopy

In order to negate differences between DnaK and its phosphoablative mutant (DnaK_Y601F), a circular dichroism spectrum of both the purified proteins was recorded. CD measurements were made on a Jasco J-810 instrument at RT. 0.1 cm path length suprasil cells were used for all the measurements. Baselines were measured with the buffer solution (50 mM NaH_2_PO_4_ pH 7.5, 100 mM NaCl) in which the proteins were dissolved. At least five repeat scans were obtained for each sample and its respective baseline. The averaged baseline spectrum was subtracted from the averaged sample spectrum. Measurements were made in the range of 240–190 nm (far UV CD). Scanning mode was continuous. The scanning speed was set to 100 nm/min with a response time being 1 s. Bandwidth was set at 1 nm.

### Protein Phosphorylation Assay

For *in vitro* phosphorylation assays, protein concentrations and incubation times in all assays are specified in figure legends. By auto-radiography, proteins were incubated at 37°C in a buffer containing 50 mM Tris pH 7.5, 100 mM NaCl, 5 mM MgCl_2_, 5% glycerol. Reactions were started by adding 50 mM ATP containing 20 mCi mmol^-1^ [γ-^32^P]-ATP and stopped by boiling at 100°C. Samples were separated on an 8–12% SDS-polyacrylamide gel. The radioactive phosphorylated proteins were detected by auto-radiography using a phosphorimager from FUJI, as described previously (Mijakovic et al., 2003). For staining purposes, proteins were incubated at 37°C in the same buffer mentioned above, using only non-radioactive 1 mM ATP. For dephosphorylation experiments, DnaK was pre-incubated with PtkA and TkmA for 1 h at 37°C before adding PtpZ. The reactions were stopped by boiling at 100°C, and samples were separated on Mini-PROTEAN^®^ TGX^TM^ Gel (BIO-RAD). Signals from phosphorylated protein were revealed by Pro-Q^®^ Diamond phosphoprotein stain (Life Technologies) as described previously (Shi et al., 2014b). Briefly, after fixing in a solution containing 50% methanol and 10% acetic acid, the gel was stained by the Pro-Q^®^ Diamond phosphoprotein stain for 90 min. Destaining was performed in a solution containing 20% ACN and 50 mM sodium acetate, pH 4.0. Tyrosine-phosphorylation of DnaK WT purified from *E. coli* was detected with biotinylated Phospho-Tyrosine Mouse mAb (P-Tyr-100; Cell Signaling). The signal was revealed with a secondary Anti-biotin Antibody, HRP-linked (Cell Signaling), using the ECL Western Blotting Detection Kit (GE Healthcare). To dephosphorylate DnaK WT, the protein was incubated with PtpZ/alkaline phosphatase (Life Technologies) at 37°C for 1 h.

### Identification of the Secondary Phosphorylation Site of DnaK by Mass Spectrometry

*In vitro* phosphorylation of DnaK WT by PtkA and TkmA was performed as described above for Pro-Q^®^ Diamond phosphoprotein staining purpose. The sample was precipitated with acetone and methanol, and then dissolved in denaturation buffer containing 6 M urea and 2 M thiourea in 10 mM Tris-HCl pH 8.0. In-solution digestion using the endoprotease Trypsin (Promega) was carried out next with 10 μg of protein, and further purified using C18 stage-tips, as described previously. Peptides were subjected to nano-LC-MS/MS measurements on the LTQ-Orbitrap Elite (Thermo Fisher Scientific).

### ATP Hydrolysis Activity Measurement

ATP hydrolysis activity was measured coupled with NADH oxidation as described by Lindsley (2001). The reaction was performed in a buffer containing 50 mM potassium -HEPES, pH 7.5, 150 mM potassium acetate, 8 mM magnesium acetate, 5 mM β-mercaptoethanol, 250 μg BSA, 2 mM phosphoenolpyruvate, 0.16 mM NADH, 5 U pyruvate kinase, 8 U lactate dehydrogenase (LDH). To measure the activity of DnaK, DnaJ, or GrpE, 2.5 μM of each protein was included in the reaction; to measure the activity of DnaK in the presence of DnaJ or/and GrpE, 0.25 μM of each protein was used. Reaction was start by adding 2 mM ATP. Enzyme kinetics was measured at A_340_ by FLUOstar Omega. ATP hydrolysis activity of each protein was expressed as ATP hydrolysis rate/moles of the protein (μM min^-1^ nmol^-1^).

For heat-resistance assay, 5 μM DnaK in a buffer containing 50 mM potassium -HEPES, pH 7.5, 150 mM potassium acetate, 8 mM magnesium acetate, 4 mM ATP was split into two aliquots. One was treated at 65°C for 5 min, the other was kept at RT. The ATP hydrolysis activity was measured for both the aliquots as described above.

### Pull Down Assay

Immunoprecipitation was carried out by Pierce Classic IP Kit. Briefly, 100 μg of DnaK and 100 μg of DnaJ was incubated with 1 μg 6x-His Epitope Tag Antibody (HIS.H8; Life technologies) in 450 μL IP Lysis/Wash Buffer. The immune complex was captured by Pierce Protein A/G Agarose and eluted in 45 μL Non-reducing Lane Marker Sample Buffer. Immunoprecipitation of DnaK with GrpE was performed using the same strategy. Fifteen microliter of the eluted sample was used for immunoblotting with anti-Strep tag (Bio-Rad), and 5 μL for that with anti-His tag (Life technologies).

### Lactate Dehydrogenase (LDH) Refolding Assay

Chemical denaturation and renaturation experiment was performed as described (Nakamoto et al., 2014). Briefly, 20 μM LDH was denatured in a buffer containing 50 mM potassium-HEPES, pH 7.5, 5 M urea and 5.0 mM DTT for 30 min at 25°C. For the refolding assay, 2 μL of denatured LDH was added to 198 μL of refolding solution containing 50 mM potassium-HEPES, pH 7.5, 150 mM potassium acetate, 10 mM magnesium acetate, 10 mM phosphoenolpyruvate, 0.16 mM NADH, 10 U pyruvate kinase, 10 mM DTT, 4 mM ATP, and 1 μM DnaK. The activity of LDH was revealed by NADH oxidation. The kinetics at A_340_ was measured by FLUOstar Omega.

### *In Vivo* Heat-Shock and Heat-Resistance Assay

*In vivo* heat-shock assay for liquid culture was carried out by splitting the culture into two, at the early exponential phase (OD_600_ 0.3). One was kept growing at 37°C for 5 min, while the other was treated at 55°C for 5 min. Both treated and untreated cultures were spread on plates by serial dilutions to obtain the number of cells that survived. For heat-resistance assay done on plates, serial dilutions of culture at OD_600_ 0.3 were spotted onto plates and incubated at 58°C for 10 h and additional 4 h at 37°C, with additional of 0.025% xylose when needed.

## Results and Discussion

### Triple SILAC-based Screening Reveals DnaK as a Substrate of PtkA and PtpZ

To identify novel substrates of the BY-kinase PtkA and the phosphotyrosine-protein phosphatase PtpZ, we performed a global and quantitative site-specific phosphoproteomics analysis of the *B. subtilis* WT, Δ*ptkA* (kinase knockout) and Δ*ptpZ* (phosphatase knockout) strains, using the triple-SILAC approach (**Supplementary Figure 1A)**. The WT (Δ*lysA*) strain was labeled with light lysine, the Δ*ptkA*, Δ*lysA* strain was labeled with medium and the Δ*ptpZ*, Δ*lysA* strain was labeled with heavy lysine. Since the phosphatase PtpZ was seen to be abundantly expressed during the stationary phase and the BY-kinase PtkA was observed to be abundant during mid-logarithmic growth phase, replicate individual experiments were performed by harvesting cultures during both stages of growth (**Supplementary Figure 1B**). Proteins extracted from the three SILAC cultures (one for each strain), which were labeled with the corresponding forms of lysine during protein biosynthesis, were mixed in equal ratios and subjected to both proteomic and phosphoproteomic analysis. The identified phosphotyrosine sites were normalized with respect to protein abundance. Normalized phosphotyrosine sites with low M/L (medium lysine/light lysine) ratio were considered as candidate substrates of PtkA, while phosphotyrosine sites with high H/L (heavy lysine/light lysine) ratio were considered as candidate substrates of PtpZ (**Supplementary Figure 1A**). Overview of the experimental setup is shown in the **Supplementary Figure 1A**.

Phosphoproteome analysis identified a total of 288 phosphorylation events, of which 170 were quantified. Two hundred and thirty-nine phosphopeptides were localized with a probability of ≥0.75 and 95 of them had a PEP of ≤0.001, which was used as a quality threshold. Ratios of protein abundance obtained from the analysis of the proteome were used to normalize the quantified phosphorylation sites. A total of 2451 proteins were identified and 2391 of them were quantified through proteomic analysis. All detected proteins and phosphorylation sites in the PtkA/PtpZ screen are listed in **Supplementary Table 1**. The incorporation of the medium and heavy label was found to be above 98% in all of the replicates (**Supplementary Figures 1C,D**).

**Table 1:** Tyrosine-phosphorylation sites detected in this study.

Protein name	Protein function	Modified sequence	Position	Δ*ptkA*/WT^a^	Δ*ptpZ*/WT^b^	Reported before
AcoB	Acetoin dehydrogenase E1 component	GEVPEDY(ph)Y(ph)TIPLGK	200			
			201			
AppA	Oligopeptide ABC transporter	Y(ph)HPKRDLY(ph)NIEK	527	0.61	0.45	
			534	0.61	0.45	
CatE	Catechol 2,3-dioxygenase	MTSIHEDTHIGY(ph)AK	12			
ComFB	Late competence	VSPSY(ph)VTDFK	46			
ComGB	Competence, DNA uptake	SGLSIY(ph)DSLNAFK	208			
CotH	Inner spore coat protein	TLQSLFTIEY(ph)MEPK	308			
DnaK	Molecular chaperone	ADDNVVDAEY(ph)EEVNDDQNK	601	0.3	1.5	
Fbp	Fructose-1,6-bisphosphatase	RAMTTFERY(ph)FIK	486	0.94	1.00	
MgsA	Methylglyoxal synthase	NHDLY(ph)ATGTTGLK	32	1.04	1.00	
PbpD	Penicillin-binding protein 4	LTELAY(ph)SY(ph)QLEKK	156			
			158			
PrpD	Methyl-*cis*-aconitase	LARPLDAY(ph)VMENVLFK	268			
RplA	Ribosomal protein L1	EAEAAGADFVGDTDY(ph)INK	100	1.19	1.07	
RsbV	Anti-anti-SigB	DVSY(ph)MDSTGLGVFVGTFK	53			Macek et al., 2007
SdpB	Maturation of the SdpC toxin	TVLFY(ph)IMTIIK	159			
ThiC	Biosynthesis of the pyrimidine moiety of thiamine	EFVDTGSNLY(ph)Q	589			Soufi et al., 2010
ThrC	Threonine synthase	SPIALVNSVNPY(ph)RIEGQK	157	0.79	0.85	
YqiB	exodeoxyribonuclease VII large subunit	MGEAAY(ph)VTVSALTK	6	1.26	1.32	
YbfP	HTH-type transcriptional regulator	MY(ph)HMPPGAY(ph)RTFMK	96	1.35	1.16	
			103	1.35	1.16	
YdfE	Uncharacterized protein	Y(ph)EAAGLTPLQSK	113			
YdgA	Uncharacterized protein	PY(ph)QINIANIK	3			
YfnD	Uncharacterized protein	MIY(ph)MPYILNLK	271			
YkoV	DNA-end-binding protein Ku	GY(ph)EY(ph)VKGKYVVLTDEDLK	81			
			83			
YlbG	Uncharacterized protein	ENRRQGMVVY(ph)LHSLK	11			
YncB	Micrococcal nuclease	DTVRY(ph)LLVDTPETKK	92			
YocA	Transposon-related protein	QFTLMY(ph)KTGK	122			
SunI	Sublancin immunity protein	Y(ph)GVADNIDYK	57	0.11	3.36	Ravikumar et al., 2014
YopU	SPBc2 prophage-derived uncharacterized protein	MNIFVDQDNY(ph)K	10			
YoqL	SPBc2 prophage-derived putative HNH endonuclease	SWLPATEENFY(ph)SVK	32			
YotM	SPBc2 prophage-derived uncharacterized protein	GY(ph)LFPFELKSTKSK	56			
YozV	Uncharacterized protein	YNFYY(ph)FQQQSK	72			
YqcE	Uncharacterized protein	MNYWVLALHY(ph)NWASSEMVK	10			
YvbJ	Uncharacterized protein	ELHQSSSY(ph)NDYTK	416			
PtkB	Protein tyrosine kinase	KSEHY(ph)SY(ph)	227	0.3	0.62	Elsholz et al., 2014
			225			
YvpB	Uncharacterized protein	YVY(ph)LNDPYGY(ph)K	217			
			224			
SasA	(p)ppGpp synthetase	NELLVY(ph)K	25			
Ugd	UDP-glucose dehydrogenase	LEESIQVLLY(ph)GQMVTRK	100	1.42	0.63	
PtkA	Protein tyrosine kinase	HSEY(ph)GY(ph)Y(ph)GTK	225	2.13	2.02	Soufi et al., 2010
			227	2.20	2.05	
			228	2.41	2.07	
YxiD	Toxin protein	EY(ph)KKMSPIETAK	463			


*Tyrosine-phosphorylation sites with quantification are shown in Δ*ptkA*/WT and Δ*ptpZ*/WT. ^a^M/L (medium lysine/light lysine) phospho-site ratio normalized to proteome, where applicable. ^b^H/L (heavy lysine/light lysine) phospho-site ratio normalized to proteome, where applicable.*

The global phosphoproteome analysis of knockout strains, followed by normalization against the proteome, led to the detection of six differentially down-regulated phosphorylation events in the Δ*ptkA* strain and four differentially up-regulated events in the Δ*ptpZ* strain (**Figure 1A**). Three phosphotyrosine sites of PtkA- Y225, Y227, and Y228, were detected as down-regulated in the Δ*ptkA* strain. They are known PtkA autophosphorylation sites (Mijakovic et al., 2003). Although these three sites were absent in the Δ*ptkA* strain, this outcome is a result of an artifact of normalization with the WT control strain. Y225, Y227, and Y228 of PtkA are also known to be dephosphorylated by PtpZ (Mijakovic et al., 2003), and they were duly detected as up-regulated in the Δ*ptpZ*, thus acting as a positive control. Phosphorylation of another *B. subtilis* BY-kinase, PtkB (YveL, EpsB), at the residue Y227, was down-regulated in the Δ*ptkA* strain. PtkB plays a role in production of exopolysaccharides (Elsholz et al., 2014) and biofilm development (Gerwig et al., 2014). It is known that some level of interplay between PtkA and PtkB exists, since the two kinases seem to be able to swap their respective transmembrane activators, TkmA and TkmB (Shi et al., 2014c). This result suggests that in addition, PtkA may be able to phosphorylate PtkB, or otherwise indirectly contribute to its autophosphorylation at the residue Y227. Interestingly, phosphorylation of PtkB was not up-regulated in the Δ*ptpZ* strain, suggesting that this phosphatase is not responsible for its dephosphorylation. Among other PtkA- and PtpZ-dependent phosphoproteins were SunI and DnaK. Phosphorylation of both SunI and DnaK was down-regulated in Δ*ptkA* and up-regulated in Δ*ptpZ*. SunI, a bacteriocin producer immunity protein that plays an important role in conferring immunity to the bacterium against sublancin, was phosphorylated at Y57. DnaK, a class-I heat-shock protein that acts as a molecular chaperone, was detected to be phosphorylated at Y601. It is important to remark that in this study we have detected 47 tyrosine-phosphorylation sites (**Table 1**), only eight of which have been detected previously. The result suggests that tyrosine phosphorylation is involved in multiple cellular functions: the development processes such as sporulation (CotH and SdpB) and biofilm formation (PtkB), competence (ComFB and ComGB), biosynthesis (AcoB, CatE, Fbp, MgsA, ThiC, ThrC, and Ugd), cellular regulation (RsbV, PtkA, YbfP, and SasA) and other physiological processes (DnaK, PbpD, RplA, YqiB, YkoV, YocA, SunI, and YxiD). Ugd, which had previously been found to be phosphorylated at Y81 by PtkA (Petranovic et al., 2009), was now found to be phosphorylated also at Y100.

Under the employed experimental conditions, we did not detect any of the previously characterized PtkA substrates as differentially regulated (Mijakovic and Deutscher, 2015). This emphasizes the transient nature of protein phosphorylation. The overlap of phosphorylation events detected in this study, where *B. subtilis* was grown in the minimal medium, with our previous study in which *B. subtilis* was grown in the rich LB medium (Macek et al., 2007), was very limited. Out of 103 phosphorylation sites detected in the LB medium (Macek et al., 2007), only 20 were detected in this study. Moreover, the dynamics of phosphorylation sites is known to change dramatically along the growth curve, and not all sites can be detected at all experimental time points, even in the same medium (Ravikumar et al., 2014). Therefore all substrates of a given kinase and phosphatase cannot be detected in a single experiment.

### DnaK Is Phosphorylated by PtkA and Dephosphorylated by PtpZ *In Vitro*

**FIGURE 2:**
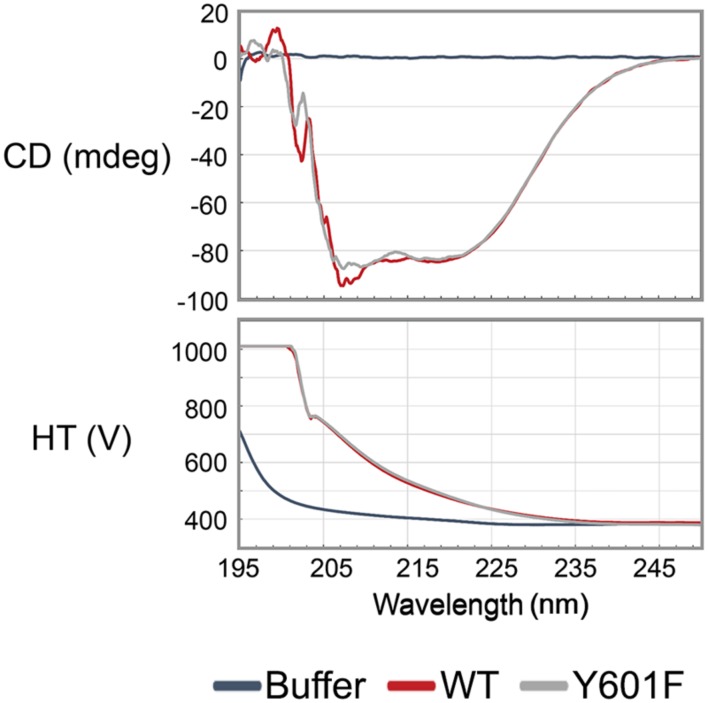
**The mutation Y601F did not affect the folding of DnaK.** Far UV Circular dichroism spectrum was measured in the range of 240 nm to 190 nm. WT: DnaK wild type, Y601F: DnaK Y601F, the mutant of DnaK in which the tyrosine (Y) at 601 was replaced by phenylalanine (F). CD, circular dichroism; HT, high tension voltage.

**FIGURE 3:**
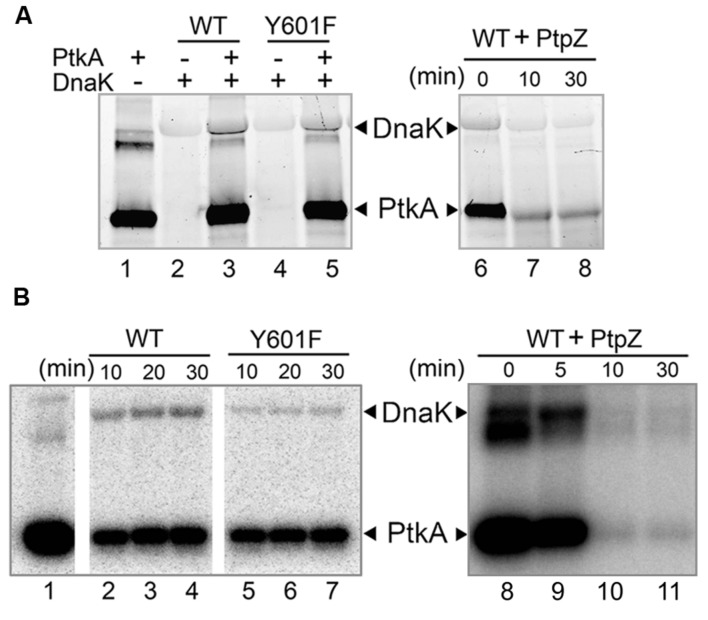
**Y601 of DnaK is phosphorylated by PtkA and dephosphorylated by PtpZ *in vitro.*** Bands corresponding to phosphorylated DnaK and PtkA are indicated by arrows. **(A)** Phosphorylation and dephosphorylation assays by Pro-Q Diamond Phosphoprotein Gel Staining. In lane 1–5, reactions were performed with 1.2 μM PtkA, 1.2 μM TkmA, 3.6 μM DnaK WT, or Y601F, and incubated for 30 min. Presence of key proteins in the assays is indicated with ± above each lane. In lanes 6–8, 5 μM PtpZ was added into pre-incubated reaction containing 0.6 μM PtkA, 0.6 μM TkmA, and 3.6 μM DnaK WT. The incubation times are given above each lane. **(B)** Autoradiography images of the phosphorylation of DnaK WT (lanes 2–4) and Y601F (lanes 5–7) in the presence of PtkA, and dephosphorylating of DnaK WT (lanes 8–11) in the presence of PtpZ. Phosphorylation assay was performed with 1 μM PtkA, 1 μM TkmA, and 3 μM DnaK. PtkA was incubated alone in the lane 1. In dephosphorylation assay, 5 μM PtpZ was added into pre-incubated reaction containing 1 μM PtkA, 1 μM TkmA, and 3 μM DnaK WT. The incubation times are given above each lane.

Following the triple SILAC screening, we set out to explore the functional relevance of one differentially phosphorylated phosphotyrosine-site, Y601 of DnaK. Manual validation of the MS/MS spectrum of the phosphorylated DnaK peptide showed good coverage and annotation of fragment ions (**Figure 1B**). In order to assess whether DnaK can be phosphorylated by PtkA and dephosphorylated by PtpZ, we proceeded with an *in vitro* phosphorylation/dephosphorylation assay. WT DnaK was heterologously expressed in *E. coli* with an N-terminal 6xHis-tag and purified. We also constructed and purified a non-phosphorylatable version of protein, DnaK Y601F, in which the phosphorylated tyrosine 601 was replaced by phenylalanine, thus removing the hydroxyl group required for phosphorylation. The mutation of Y601F did not affect the folding of DnaK, as demonstrated by very similar circular dichroism spectra of the DnaK WT and Y601F (**Figure 2**). We therefore concluded that the mutation is structure-neutral, and can be used to mimic the non-phosphorylated state of the protein. Pro-Q^®^ Diamond phosphoprotein staining suggested that both DnaK and DnaK Y601F were to some extent phosphorylated during heterologous expression in *E. coli* (**Figure 3A**). This was confirmed by an independent method, Western blotting with an anti-phosphotyrosine antibody (**Supplementary Figure 2**). When incubated with PtkA and ATP, the phosphorylation signal increased both for DnaK and DnaK Y601F, and PtpZ was able to remove the phosphorylation signal (**Figure 3A**). This confirmed that DnaK can be phosphorylated by PtkA and dephosphorylated by PtpZ, but left some doubts as to the identity of the phosphorylated residue. To differentiate between the pre-existing phosphorylation of DnaK and its phosphorylation catalyzed by PtkA, we performed a phosphorylation assay based on incorporation of ^32^P from ^32^P-gamma-labeled ATP (**Figure 3B**). Here it was clear that phosphorylation of DnaK was more efficient than that of DnaK Y601F. However, some residual phosphorylation of DnaK Y601F occurred on residue(s) other than Y601, consistent with the results of Pro-Q^®^ Diamond phosphoprotein staining. Using mass spectrometry, we identified another DnaK residue phosphorylated by PtkA *in vitro*: the tyrosine 573 (**Supplementary Figure 3**). We concluded that Y601 is the main residue phosphorylated by PtkA, and Y573 is the secondary phosphorylation site.

### The Mutation on Tyrosine 601 of DnaK Impairs Its Interaction with Co-chaperones DnaJ and GrpE

**FIGURE 4:**
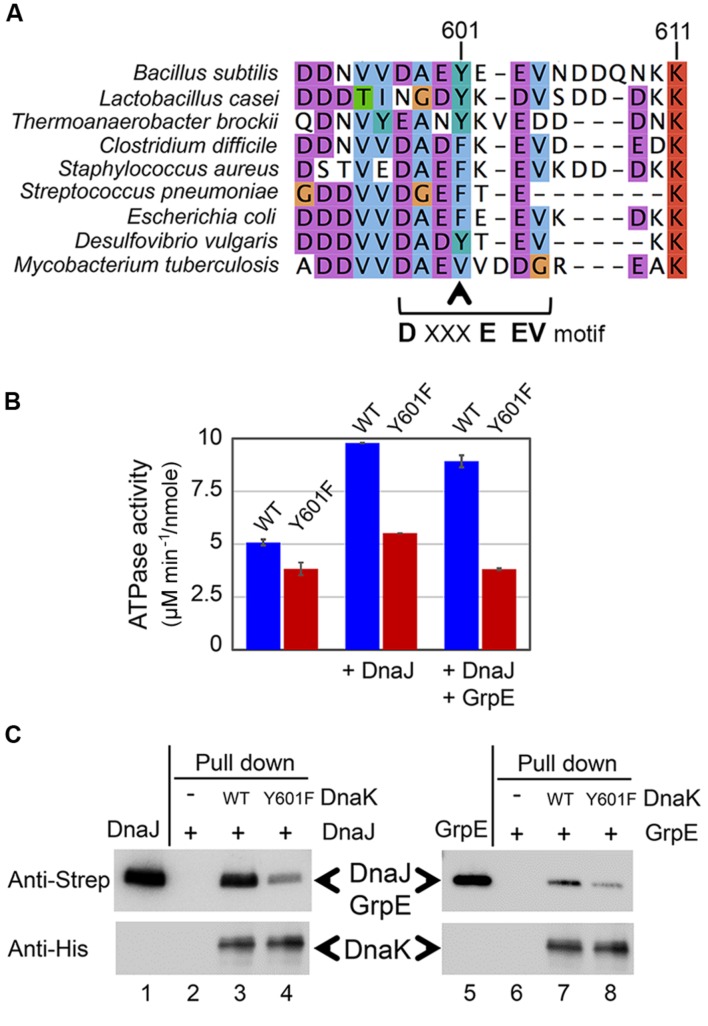
**DnaK Y601F exhibited impaired interaction with its co-chaperone proteins DnaJ and GrpE.** **(A)** Sequence alignment of DnaKs from *Bacillus subtilis*, *Lactobacillus casei, Thermoanaerobacter brockii, Clostridium difficile, Staphylococcus aureus, Streptococcus pneumonia, Escherichia coli, Desulfovibrio vulgaris*, and *Mycobacterium tuberculosis*. Y601 in the *B. subtilis* DnaK is indicated by the arrow. **(B)** ATPase activity of DnaK WT and Y601F in three conditions: incubated alone, in the presence of DnaJ, and in the presence of both DnaJ and GrpE. The ATP hydrolysis activity of DnaJ and GrpE was subtracted from all relevant samples. The results are the mean values from five independent replicates, with error bars representing the standard deviation. ATPase activity is expressed as ATP hydrolysis rate/moles of the protein (μM min^-1^/nmol). **(C)** Interaction of DnaK WT/Y601F with DnaJ and GrpE detected by a pull-down assay. Experiment was performed with Strep-tagged DnaJ and GrpE, and 6xHis-tagged DnaK. DnaJ (lane 1) and GrpE (lane 5) were loaded directly on SDS-PAGE. DnaJ or GrpE incubated without DnaK (lanes 2 and 6), with DnaK WT (lanes 3 and 7), and with DnaK Y601F (lanes 4 and 8) were subjected to immunoprecipitation with an anti-6xHis antibody. The eluates from different samples were separated by SDS-PAGE, and detected by immunoblotting with anti-Strep tag and anti-His tag antibodies. The experiment was done in duplicates, and a representative image is shown.

The C-terminus of DnaK is known to be important for its interaction with its co-chaperone DnaJ (Gao et al., 2012). More specifically, the C-terminal helical subdomain downstream of the substrate-binding domain of DnaK is important for DnaJ binding, since the protein lacking this domain is unable to bind DnaJ. Recently, a conserved motif DXXXEEV was identified in the extreme C-terminal tail of bacterial DnaKs. In *E. coli*, this motif was shown to be crucial for *in vivo* cell survival upon heat shock and *in vitro* chaperone activity of DnaK. Moreover, the DXXXEEV motif was predicted to be a potential protein binding region (Smock et al., 2011). Interestingly, Y601 in *B. subtilis* DnaK is situated within this motif (**Figure 4A**). We examined 934 available sequences of bacterial DnaKs, and found Y601 to be conserved in 20.88% of them, and in 25.35% of DnaKs from Firmicutes. The most common residue at this position is phenylalanine, which is a non-phosphorylatable version of tyrosine. Phenylalanine is found at the position equivalent to *B. subtilis* Y601 in 50.43% of all bacterial DnaKs, and 66.55% of DnaKs from Firmicutes. Reversible phosphorylation of tyrosine Y601 adds a negatively charged residue in this DXXXEEV motif (DAEYEEV in *B. subtilis*), and could thus be expected to have an impact on DnaK function. One of the known consequences of DnaK interaction with DnaJ is the enhancement of the ATP hydrolysis catalyzed by DnaK (Laufen et al., 1999). Therefore we asked whether DnaJ would be capable of enhancing the ATPase activity of DnaK Y601F. As shown in the **Figure 4B**, DnaK Y601F exhibited a slightly impaired ATPase activity compared to the WT. More interestingly, neither DnaJ, or DnaJ and GrpE together, provided any detectable enhancement of the ATPase activity of DnaK Y601F. Since DnaJ and GrpE stimulate DnaK ATPase activity via a direct protein–protein interaction, DnaK Y601F should be expected to have an impaired interaction with its co-chaperones. To test the interaction of DnaK with DnaJ and GrpE we used a pull-down assay (**Figure 4C**). Binary interactions of DnaK with DnaJ and GrpE were readily detectable. Mutated protein DnaK Y601F had a clearly diminished capacity to interact with both co-chaperones, indicating that residue tyrosine 601 indeed contributes to the interactions. Thus, we concluded that tyrosine 601 of DnaK is important to its interaction with co-chaperones DnaJ and GrpE, and consequently to activation of its ATPase function.

### DnaK Y601F Exhibited Diminished Chaperone Activity

**FIGURE 5:**
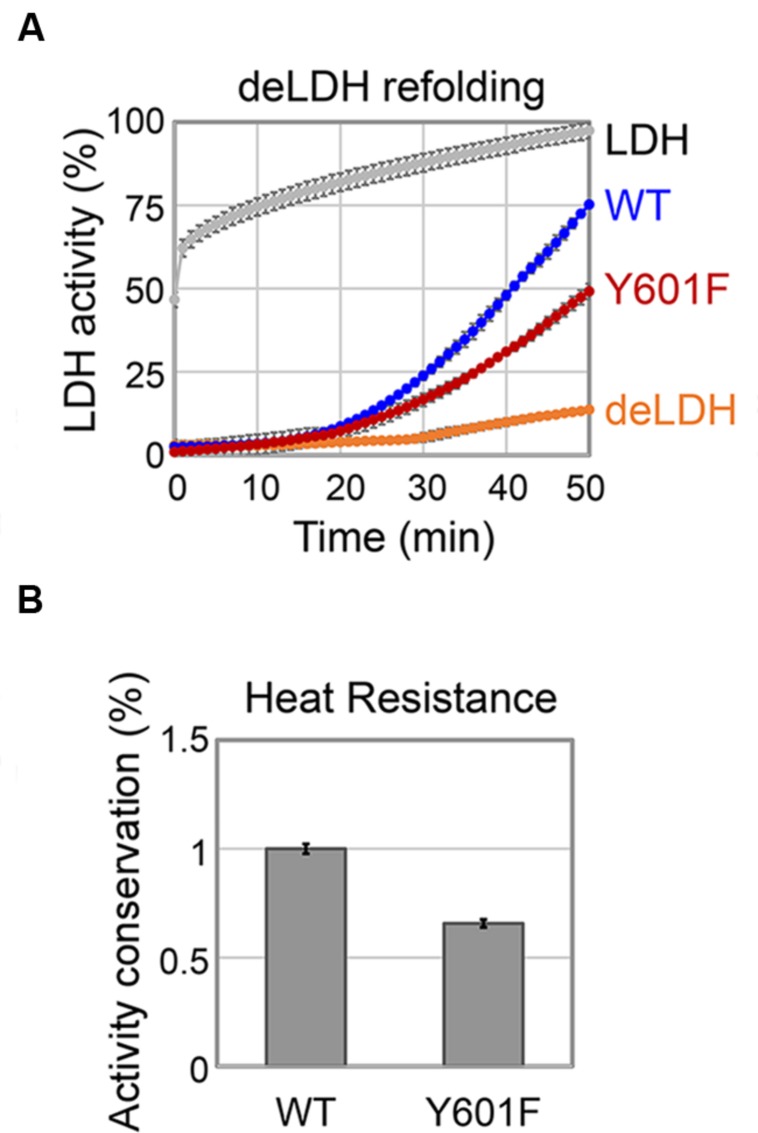
**DnaK Y601F is less efficient in chaperone activity.** **(A)** Refolding of GuHCl-denatured LDH by DnaK WT and Y601F. LDH: activity of LDH without GuHCl treatment; WT: activity of denatured LDH in the presence of DnaK WT; Y601F: activity of denatured LDH in the presence of DnaK Y601F; deLDH: activity of denatured LDH in the absence of DnaK. **(B)** Heat resistance of DnaK WT and Y601F to a heat treatment at 65°C for 5 min. DnaK ATP hydrolysis activity is expressed as a ratio of activity after and before the heat treatment. DnaK Y601F was normalized with respect to the WT. The results are the mean values from five independent replicates, with error bars representing the standard deviation.

One of the major roles of molecular chaperones is to facilitate refolding of denatured proteins at high temperatures. Based on the *in vitro* data presented in the previous section, we concluded that DnaK tyrosine 601 is crucial for its interaction with co-chaperones and its ATPase function, both of which contribute to its activity as a molecular chaperone. Therefore, we asked whether the mutation on tyrosine 601 would have a measurable impact on its ability to re-fold denatured proteins. To test this, we established an assay with LDH denatured with a chaotropic agent. The activity of DnaK was measured as its capacity to restore the enzyme activity of denatured LDH (**Figure 5A**). WT DnaK, was able to restore 75% of the original activity of the LDH in 50 min. The Y601F mutant of DnaK was less efficient, and restored only about 50% of the original LDH activity in the same period. Thus, we concluded that the tyrosine 601 is important for the maintenance of the DnaK chaperone activity. In order to accomplish their role, molecular chaperones must themselves remain active under denaturing conditions. Therefore, we asked whether the DnaK Y601F remains stable under heat shock. To answer this, we measured the conservation of the ATPase activity of DnaK at high temperature *in vitro* (**Figure 5B**). The results are expressed as the ratio of ATPase activity of the heat-treated protein to the non-treated protein. DnaK Y601F exhibited a more severe loss of activity than the WT, indicating an impaired capacity to tolerate high temperature. We hypothesize that the Y601 could be involved in the regulation of DnaK chaperone activity through its phosphorylation. Previous findings in *E. coli* suggested chaperones became phosphorylated during heat shock (Sherman and Goldberg, 1992, 1993). The phosphorylated fraction of *E. coli* chaperones exhibited an increased capacity to interact with and re-fold denatured proteins. This is in agreement with the fact that DnaK WT, partially phosphorylated during production in *E. coli*, shows higher chaperone activity. However, we could not exclude the possibility that the impaired chaperone function of DnaK Y601F was caused by the lack of the free hydroxyl group, rather than by phosphorylation itself. An example of such a scenario is phosphorylation of the *B. subtilis* FatR, a transcriptional regulator involved in regulation of polyunsaturated fatty acids metabolism. FatR forms dimers, which bind the target DNA. The hydroxyl group of its tyrosine 45 is essential for the protein–DNA interaction, so phosphorylation basically had the same effect as removing the hydroxyl by replacing tyrosine 45t with phenylalanine, namely the loss of interaction with DNA (Derouiche et al., 2013).

### *B. Subtilis* DnaK Y601F Strain Shows Less Cellular Survival upon Heat Shock

**FIGURE 6:**
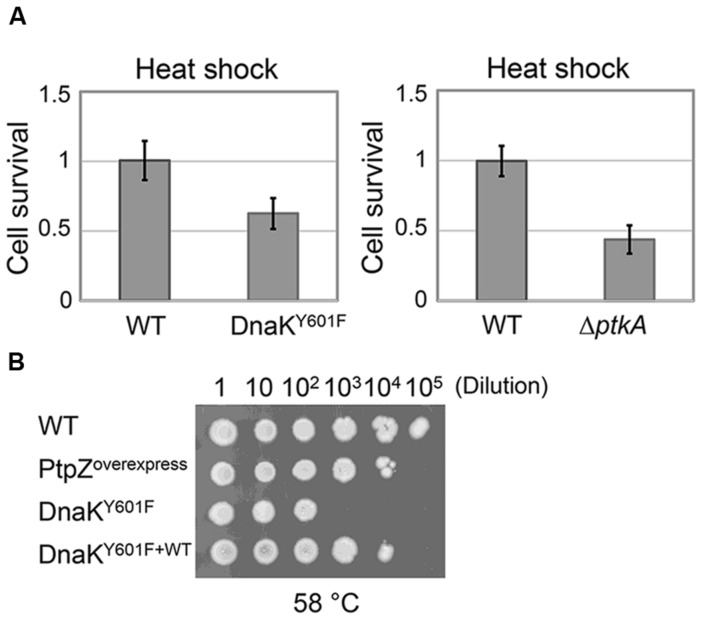
**Non-phosphorylated state of DnaK results in impaired heat resistance *in vivo*.** **(A)** Survival of the heat shock treatment (55°C for 5 min) of *B. subtilis* strains Δ*ptkA*Δ*lysA* and *dnaK* Y601F compared to their respective WT. Heat resistance is expressed as the number of CFUs after treatment/number of CFUs without treatment. Each strain was normalized with respect to the WT. The results are the mean values from three independent replicates, with error bars representing the standard deviation. **(B)** Growth tests for *B. subtilis* strain: WT, *ptpZ* overexpression, Y601F and Y601F carrying WT copy of *dnaK*. Serial dilutions of each culture were spotted onto plates and incubated at 58°C for 10 h and additional 4 h at 37°C. The result is representative of three independent replicates.

In order to investigate the effect of DnaK tyrosine 601 *in vivo*, we subjected *dnaK* Y601F (point mutation at the locus) to a 5 min heat shock at 55°C, and measured the fraction of surviving cells. Compared to the WT strain, *dnaK* Y601F provoked a significant drop in survival (**Figure 6A**). The result indicated the function importance of DnaK tyrosine 601 *in vivo*. The involvement of the tyrosine 601 in DnaK function could be trigger by the hydroxyl group itself, or alternatively trough phosphorylation. We subjected the strains Δ*ptkA*Δ*lysA* (used for the triple SILAC experiment) to the same heat resistance assay. The inactivation of PtkA led to a reduced survival upon heat shock *in vivo* (**Figure 6A**), which suggests the regulatory potential of this phosphorylation event. The phosphoproteome and *in vitro* dephosphorylation data suggested that PtpZ is the phosphatase responsible for DnaK dephosphorylation (**Figures 1A** and **2A**). To test this assumption *in vivo*, we overproduced PtpZ. The strain overproducing PtpZ showed decreased ability to grow at elevated temperature (**Figure 6B**), consistent with it having less phosphorylated DnaK. In the same assay, the strain with the *dnaK* Y601F mutation had a more severe phenotype, which suggests that overexpressed PtpZ could not entirely dephosphorylate the tyrosine 601 *in vivo*. The severe phenotype of the *dnaK* Y601F mutation could be complemented by two orders of magnitude (in terms of survival/dilution) by expressing the WT *dnaK* allele ectopically (**Figure 6B**). These results suggested that the tyrosine 601 of DnaK is crucial for the cellular survival upon heat shock, and the most likely mechanism of its contribution to DnaK chaperone function is through PtkA-dependent phosphorylation and PtpZ-dependent dephosphorylation.

## Concluding Remarks

In this study, we have employed the triple-SILAC based quantitative phosphoproteomics approach to identify substrates of *B. subtilis* PtkA and PtpZ *in vivo*. We selected one differentially regulated phosphorylation site, Y601 of DnaK, to demonstrate the physiological relevance of the results obtained by this approach. We found out that the replacement of tyrosine 601 to phenylalanine leads to less interaction with its co-chaperone proteins DnaJ and GrpE, lower efficient ATPase activity and capacity to assist folding of denatured proteins and increased thermal resistance of DnaK itself *in vitro*. By impairing all of these inter-related features of DnaK, this replacement event leads to a significantly diminished survival upon heat shock *in vivo*. DnaK was found to be modified by phosphorylation also in other bacteria, such as *E. coli*. Although there is evidence indicated phosphorylation enhanced *E. coli* DnaK chaperone activity (Sherman and Goldberg, 1993), the exact mechanism of the phosphorylation-based activation has not been characterized. Our study provides the possibility that *B. subtilis* DnaK also undergo the similar regulation via phosphorylation. Given the presence of the Y601 at this particular position in only 20–25% of bacterial DnaKs (with the most common amino acid being phenylalanine), it is plausible to presume that a mutation of phenylalanine to tyrosine provided some bacteria with additional regulatory potential to control the chaperone activity. *E. coli*, for example, has phenylalanine in the equivalent position, so the phosphorylation of its DnaK during heat shock evidently occurs on a different residue. The *B. subtilis* and *E. coli* DnaKs are phosphorylated at different residues, and presumably trigged by different regulatory mechanisms represents as interesting example of convergent evolution. There seems to exist a clear selective advantage of having the chaperone activity triggered by phosphorylation. But the evolutionary paths taken by each bacterial organism to address this selective advantage differ in detailed arrangements.

## Author Contributions

LS, VR, and AD performed the experiments, LS, VR, BM, and IM carried out experimental design and data analysis, LS, VR, BM, and IM wrote the manuscript.

## Conflict of Interest Statement

The authors declare that the research was conducted in the absence of any commercial or financial relationships that could be construed as a potential conflict of interest.
