# Supplementary material for: Tyrosine 601 of Bacillus subtilis DnaK Undergoes Phosphorylation and Is Crucial for Chaperone Activity and Heat Shock Survival
Source: Front Microbiol. 2016 Apr 19;7:533. doi: 10.3389/fmicb.2016.00533 (PMC4835898; doi:10.3389/fmicb.2016.00533)
Supplement: Supplementary file 1 [file Data_Sheet_1.PDF]

Supplementary Material

Tyrosine 601 of *Bacillus subtilis* DnaK undergoes phosphorylation and is crucial for chaperone activity and heat shock survival

Lei Shi[1#], Vaishnavi Ravikumar[2#], Abderahmane Derouiche[1], Boris Macek[2*], Ivan Mijakovic[1*]

[1] Division of Systems & Synthetic Biology, Department of Biology and Biological Engineering, Chalmers University of Technology, Kemivägen 10, 41296 Gothenburg, Sweden

[2] Proteome Center Tuebingen, Interfaculty Institute for Cell Biology, University of Tuebingen, Auf der Morgenstelle 15, 72076 Tuebingen, Germany

[#]Equally contributing first authors

*** Equally contributing corresponding authors:**

Ivan Mijakovic, Division of Systems & Synthetic Biology, Department of Biology and Biological Engineering, Chalmers University of Technology, Kemivägen 10, 41296 Gothenburg, Sweden. ivan.mijakovic@chalmers.se

Boris Macek, Proteome Center Tuebingen, Interfaculty Institute for Cell Biology, University of Tuebingen, Auf der Morgenstelle 15, 72076 Tuebingen, Germany. boris.macek@uni-tuebingen.de

1 Supplementary Figures and Tables

1.1 Supplementary Figures

A

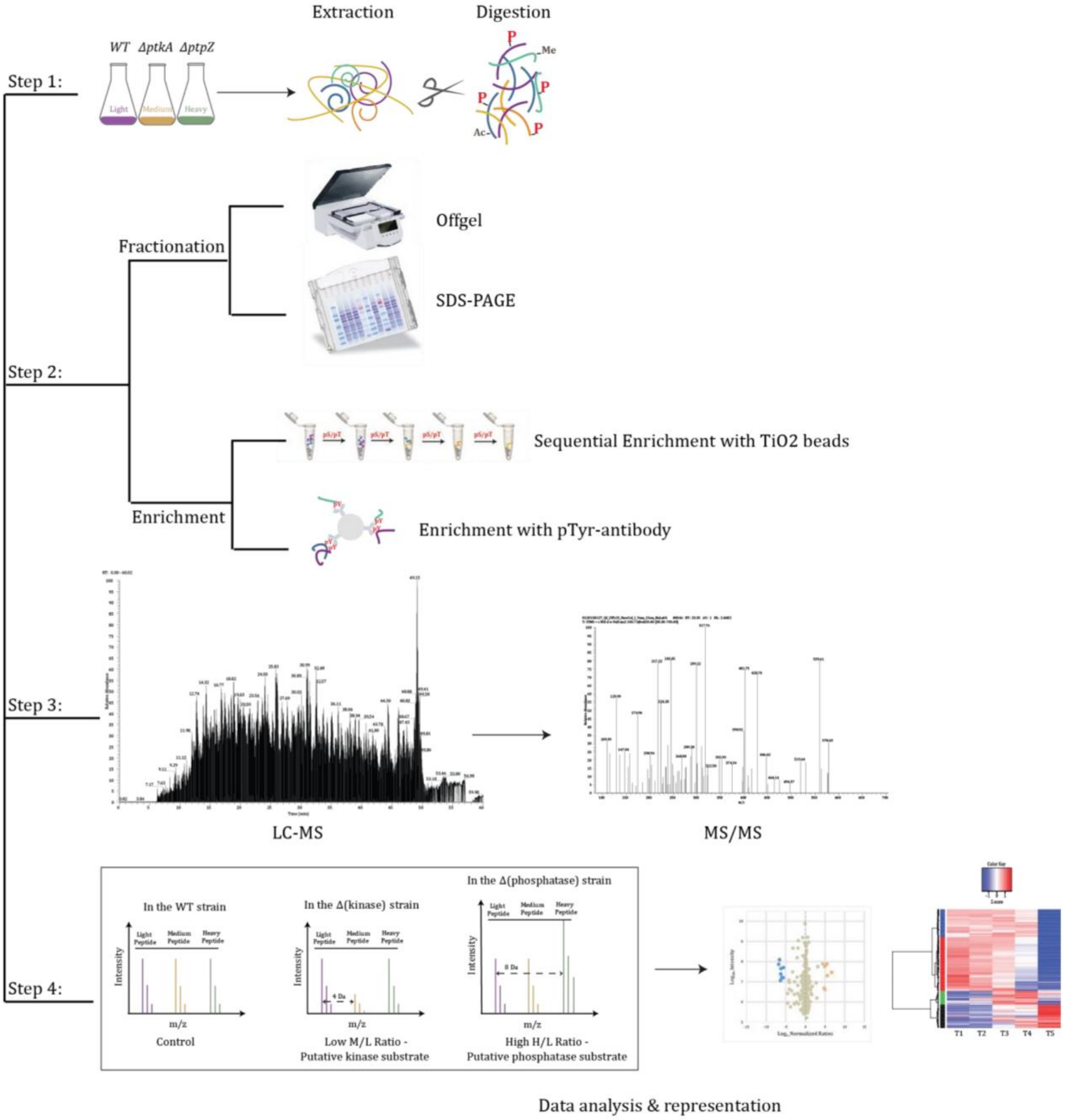

Extraction
Digestion
WT ΔptkA ΔptpZ
Step 1:
Light
Medium
Heavy
Offgel
Fractionation
SDS-PAGE
Step 2:
Sequential Enrichment with TiO2 beads
Enrichment
Enrichment with pTyr-antibody
Step 3:
LC-MS
MS/MS
In the WT strain
In the Δ(kinase) strain
In the Δ(phosphatase) strain
Light Peptide
Medium Peptide
Heavy Peptide
Intensity
m/z
Control
4 Da
Low M/L Ratio - Putative kinase substrate
8 Da
High H/L Ratio - Putative phosphatase substrate
Step 4:
T1
T2
T3
T4
T5
Data analysis & representation

B

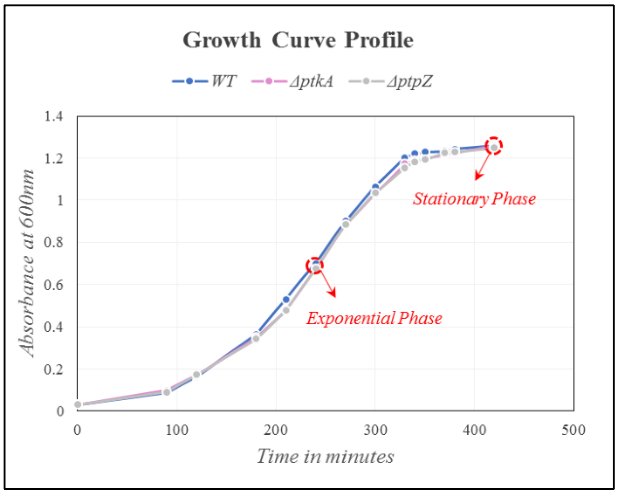

Growth Curve Profile
WT
ΔptkA
ΔptpZ
Absorbance at 600nm
Time in minutes
Stationary Phase
Exponential Phase
0
0.2
0.4
0.6
0.8
1
1.2
1.4
0
100
200
300
400
500

C

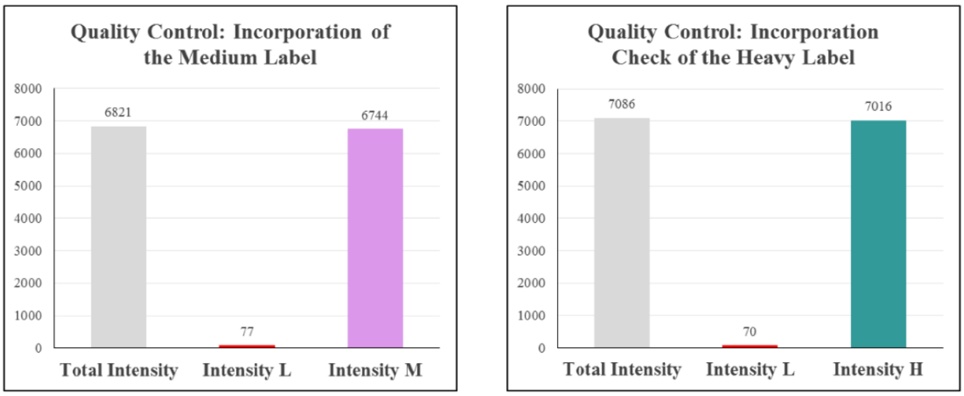

D

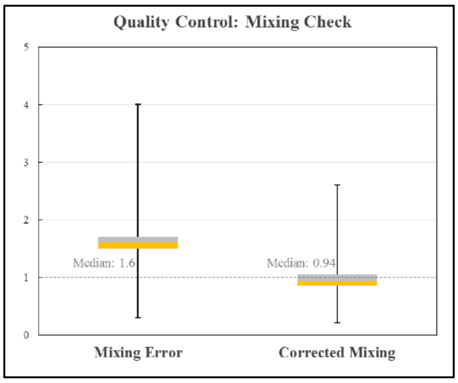

Supplementary Figure 1. SILAC-Based Screen for the identification of substrates for the bacterial-tyrosine kinase PtkA and phosphatase PtpZ. (A) Overview of the proteomics workflow adopted for the identification of novel substrates for the PtkA and PtpZ. Cells were harvested at appropriate time points. Extracted proteins were digested and fractionated for proteome analysis. Portion of the digested peptides was enriched for phosphopeptides. Wild type strain (WT) acts as a control and all ratios were normalized to this state. Proteins with a down regulated M/L ratio and/or an up regulated H/L ratio were considered as potential candidates for PtkA and PtpZ respectively. (B) Growth curve of *B.subtilis* WT (blue), Δ*ptkA* (pink) and Δ*ptpZ* (grey) strains in minimal media. The points of harvest (exponential and stationary phase) are marked by red dashed circles. (C) Quality control of the proteomics analysis. Rate of incorporation of the medium and heavy label was checked for all biological replicates. Shown here, is a single representative example, wherein, the left and the right panels represent bar plots showing > 98 % incorporation of the medium label and heavy label, respectively. Label incorporation is calculated based on the unnormalized peptide evidences having intensities above zero. (D) Quality control of the proteomics analysis. Boxplot representation of the mixing check, which was done based on the protein concentrations measured by Bradford (left), and mixing check, which was corrected based on the median of the unnormalized evidence ratios (right).

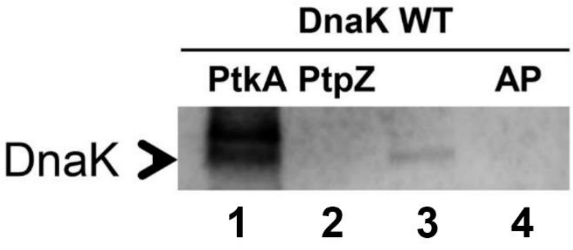

Supplementary Figure 2. DnaK wild type was partially phosphorylated during production in *E. coli.* All lanes contain 2 µM DnaK WT purified from *E. coli*. The untreated sample is shown in lane 3. In lane 1, DnaK was firther phosphorylated in the presence of 0.2 µM PtkA and 0.2 µM TkmA. In lanes 2 and 4, DnaK was dephosphorylated in the presence of 6 µM PtpZ and 10 U of alkaline phosphatase (AP), respectively. Reactions in lanes 1, 2 and 4 were incubated at 37 °C for 1 hr. Bands corresponding to phosphorylated DnaK are indicated by the arrow.

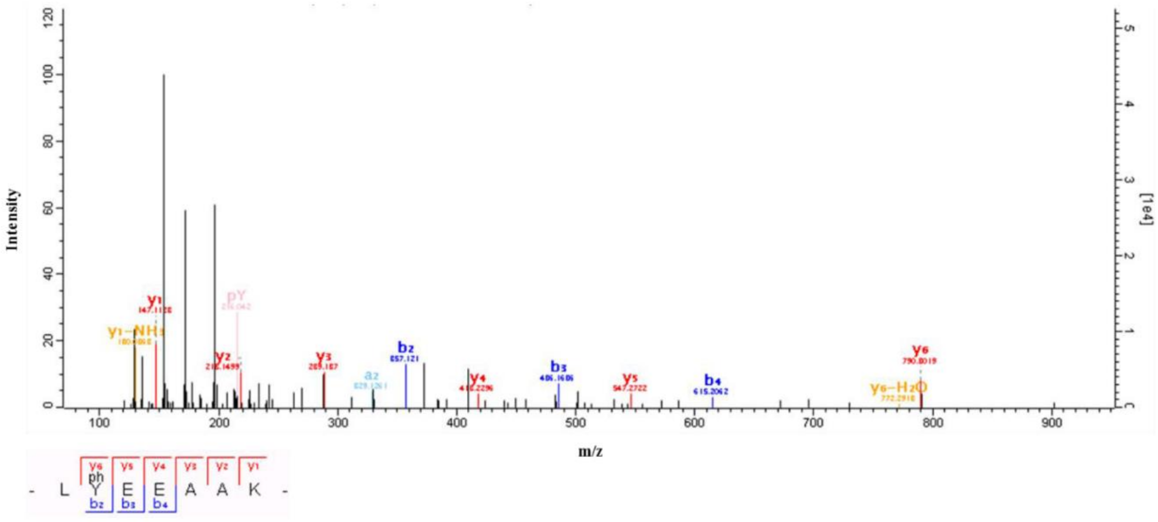

Supplementary Figure 3. DnaK is phosphorylated at tyrosine 573 by PtkA. MS/MS spectrum of the DnaK peptide depicting a secondary site of phosphorylation at Y573 during *in vitro* phosphorylation experiments.

1.2 Supplementary Tables

Supplementary Table 1. Proteome and phosphoproteome data from the SILAC-Based Screen. This table is provided as a separate Excel file.

Supplementary Table 2. PCR primers used in this study.

Primer	Sequence
**ptkAko_1**	ccggaattcattcaactcagattctggtc
**ptkAko_2**	tccccccgggaatgtccactccccgtttttc
**ptkAko_3**	tccccccgggataacgtgcatcgtgcccgt
**ptkAko_4**	cgcggatccagccggagagaaaatgctttc
**ptpZko_1**	ccggaattcgggaaggaaaatcaacaacg
**ptpZko_2**	tccccccggggtcctagccccctttttcgc
**ptpZko_3**	tccccccgggcagccgattctcatttcacc
**ptpZko_4**	cgcggatcctaatcagtccattaatcaaatc
**dnaKF_1**	cagcgtttgaaagacgcagc
**dnaKF_2**	ccatagcctctgacattttgaac
**dnaKF_3**	gagctcgaattcactggccgtcgcgacgctgaatttgaagaagtaaacgacgacc
**dnaKF_4**	cgacctgcaggcatgcaagctgtttacttcttcaaattcagcgtcgacaacg
**dnaK_ 1729_fwd**	cggggtaccatgagtaaagttatcggaatcgac
**dnaK_1729_Rev**	ccgctcgagttattttttgttttggtcgtcgtttac
**ptpZ_1729_fwd**	cggggtaccatgatcgatattcactgtcacattc
**ptpZ_1729_Rev**	ccgctcgagttaaaagaaaccaaacaattttcttct
**DnaK_fwd**	cgcggatccatgagtaaagttatcggaatcgacttaggaacaacaaactcatg
**DnaK_rev**	attcccgggttattttttgttttggtcgtcgtttacttcttcgtattcagcgtcgac
**DanK_601F_rev**	attcccgggttattttttgttttggtcgtcgtttacttcttcgaattcagcgtcgac
**DnaJ_fwd**	cgcggatccatgagtaagcgtgattactatg
**DnaJ_rev**	aaaactgcagttaatcgcctttaaacgcgcgt
**grpE_fwd**	cgcggatccatgtcagaagaaaaacaaaccgt
**grpE_rev**	aaaactgcagttattgattcactttgaccatg
